# Public acceptance of coercive measures in Nigerian mental health care

**DOI:** 10.1177/00207640241296050

**Published:** 2024-11-13

**Authors:** Deborah Oyine Aluh, Daniel Ifeanyichukwu Agu, Wisdom Joe Igbokwe, Ifunanya Genevieve Anunwa

**Affiliations:** 1 Lisbon Institute of Global Mental Health, Comprehensive Health Research Centre/NOVA Medical School, Universidade NOVA de Lisboa, Portugal; 2 Department of Clinical Pharmacy and Pharmacy Management, University of Nigeria Nsukka, Enugu, Nigeria

**Keywords:** Coercion, Nigeria, public, stigma, mental health care, mental health legislation

## Abstract

**Background:**

For the first time, Nigeria has enacted a new mental health law that regulates the use of coercive measures in mental health care.

**Aim:**

The study aimed to investigate the extent to which the Nigerian public accepts the use of coercive measures in the treatment of people with mental health conditions and to understand the impact of stigma and other sociodemographic characteristics.

**Methods:**

A cross-sectional survey was conducted among 615 adult respondents from Nigeria’s six geopolitical zones. The study instrument included a case-specific vignette, a social distance scale, and a brief sociodemographic form. Descriptive and inferential statistics were conducted with SPSS v.25 software.

**Results:**

More than half of the study respondents agreed that the vignette character should be forced to go to the hospital if he refuses to go (65%, *n* = 400), and he should be forced to take medications at the psychiatric hospital (55.1%, *n* = 339). The least accepted coercive measure was Isolation (28.8%, *n* = 177). There were significant associations between social distance and the acceptance of involuntary admissions, forced medication, mechanical restraints, and isolation (*p* < .05). Social distance score was highest among respondents who agreed that the vignette character should be isolated (24.023 ± 5.503; *F* = 24.672, *p* < .001).

**Conclusions:**

The study highlights variations in public attitudes toward coercive psychiatric measures, within the Nigerian context compared to other countries. The lower acceptance rates for isolation as a coercive measure underscore the cultural importance of social interaction in Nigeria. The relatively recent implementation of Nigeria’s Mental Health Act also suggests a potential gap in public knowledge regarding the criteria for coercive measures. Future research should aim to include diverse populations and consider longitudinal approaches to assess changes in public attitudes as awareness of mental health legislation increases.

## Introduction

The use of coercion in mental health care has long been a source of contention and debate, and it has recently received more attention following the implementation of the United Nations Convention on the Rights of Persons with Disabilities (UNCRPD) in 2008. Coercive measures such as involuntary admission, physical, mechanical, and chemical restraints, as well as seclusions, fall into the category of formal coercive measures since they are typically regulated by mental health laws in each country. Emphasizing autonomy as its core principle, the UNCRPD urges countries to revise their mental health legislation to fully safeguard and promote the fundamental human rights of people with disabilities, including those with mental health conditions. It advocates for a human-rights-based approach to mental health care, condemning involuntary care and the use of all coercive measures during treatment (Herrman et al., 2022). There is an ongoing global movement to reduce the current reliance on coercive measures in the care of persons with mental health conditions (PMHCs).

While advocating for human rights remains crucial, it must compete with numerous other urgent issues. Unfortunately, human rights, especially for specific populations such as PMHCs, are not always prioritized on the public agenda (Simmons, 2014). The movement for the rights of PMHCs has a complex and evolving history, closely intertwined with broader disability rights movements. Although the disability rights movement has been in progress since the aftermath of World War II, promoted by the substantial number of veterans with disabilities and the polio epidemic, regional advocacies have developed at a later stage. On an international scale, the UNCRPD was ratified by numerous African countries, including Nigeria. However, it is worth noting that the specific protocol to the African Charter on Human and People’s Rights addressing the rights of persons with disabilities in Africa was established more recently in 2018 (*Protocol to the African Charter on Human and Peoples’ Rights on the Rights of Persons with Disabilities in Africa |* African Union, 2018 ). Similarly, in Nigeria, the Discrimination Against Persons with Disabilities (Prohibition) Act was passed in 2019 (Discrimination Against Persons with Disabilities (Prohibition) Act, 2018), signaling a growing acknowledgment of the importance of protecting the rights of people with disabilities. Nigeria’s National Mental Health Act (National Mental Health Act, 2021), which replaced the colonial Lunacy Act of 1958 (Lunacy Act, 1958), was passed in January 2023, reflecting the country’s belated recognition of the rights of PMHCs. The new law has been commended for being more aligned with international human rights conventions (Aluh, Onu, & Almeida, 2023). It clearly outlines the rights of PMHCs, including their right to receive adequate care, participate in treatment decisions, and live free from discrimination. The Act also establishes robust safeguards to prevent abuses, setting out clear criteria for the use of coercive measures as a last resort. These measures include involuntary admissions, as well as the use of physical, mechanical, and chemical restraints (National Mental Health Act, 2021).

Despite Nigeria’s ratification of various international human rights agreements, the state of human rights has been on a decline for its citizens. Multiple armed groups are causing havoc in Nigeria, with bandits terrorizing the Northwest and the Islamic State West Africa Province (ISWAP) launching attacks in the Northeast. Agrarian conflicts between farmers and herders in the Middle Belt and Northcentral region are escalating due to ethnic and religious tensions. Security forces have been implicated in human rights abuses, including indiscriminate airstrikes, while accountability measures have been lacking. The removal of fuel subsidies and other factors has led to high inflation rates, worsening poverty, and inequalities (Human Rights Watch, 2024). Corresponding severe human rights violations against PMHCs have been reported in the community, prayer camps, traditional healing centers, and some psychiatric facilities (Human Rights Watch, 2019). A study conducted in two Nigerian psychiatric hospitals revealed significant human rights violations, including the chaining of patients to prevent absconding and the use of physical punishment for refusing medication (Aluh et al., 2022).

Previous research has highlighted the significance of stigma in influencing the acceptance of coercive measures in the treatment of PMHCs (Lauber et al., 2002; Steiger et al., 2022). In Nigeria, multiple studies conducted among diverse populations in various regions of the country have consistently shown poor mental health literacy(Aluh et al., 2018; Gureje et al., 2005) and high levels of stigma (Audu et al., 2013; James et al., 2012; Ronzoni et al., 2010; Sheikh et al., 2015; Ubaka et al., 2018). Given that coercion is a last resort measure to manage dangerousness and that stigmatization of PMHCs is linked to the attribution of dangerousness (Jorm et al., 2012), it has been hypothesized that greater stigmatization could be linked to a greater population acceptance of coercive measures (Steiger et al., 2022). This may be particularly so in Nigeria, where mental health care is primarily funded out of pocket, social services are scarce, and collectivism is deeply ingrained. By exploring the extent of acceptance and understanding the underlying factors contributing to the acceptance of coercive measures, informed interventions and policies can be developed and tailored to the context. The study’s overarching goal was to investigate the extent to which the Nigerian public accepts the use of coercive measures in the care of PMHC and to understand the impact of stigma and other sociodemographic characteristics on this acceptance.

## Methods

### Study design and participants

The study employed a cross-sectional design and was conducted among a conveniently selected sample. The sample size was computed using the Cochran formula for sample size determination (Cochran, 1963).

$$n_{0}\;=\;\frac{z^{2}.p.\;{\left(1-p\right)}^{96}}{e^{2}}$$

where:

*n*_0_ = sample size

*Z* = *Z*-score (which corresponds to the desired confidence level; 1.96 for 95% confidence level)

*p* = estimated proportion of the population with the characteristic of interest 0.5

*e* = is the desired level of precision (margin of error; typically, .05 for 5%).

The sample size was computed to be 384. More respondents were recruited to account for attrition, increased statistical power, and external validity of the study findings. A total of 615 adults 18 years old or older who were willing to participate in the survey were consecutively recruited between February 2024 and April 2024. The questionnaires were available in online and paper formats and were administered through various channels including social media, hospitals, and churches.

### Measures

The study instrument consisted of three sections. The first section presented a culturally sensitive fictitious vignette depicting a person with a first episode of psychosis and social withdrawal. This vignette was adapted from an already established vignette, first developed by Jorm et al. (1997). The adapted vignette had previously been used within the study population to assess mental health literacy (Aluh et al., 2019) and modifications were made to align the vignette with the current study’s objectives. The National Youth Corps Service (NYSC) is a mandatory post-graduate program for all Nigerian graduates. It was added to the case study for relatability. ‘*John is 24 and lives at home with his parents. He just completed his NYSC service but is now unemployed. Over the last six months, he has stopped seeing his friends and has begun locking himself in his bedroom and refusing to eat with the family or to have a bath. His parents also hear him walking about his bedroom at night while they are in bed. Even though they know he is alone, they have heard him shouting and arguing as if someone else is there. When they try to encourage him to do more things, he whispers that he won’t leave home because he is being spied upon by the neighbor. They realize he is not abusing any drugs because he never sees anyone or goes anywhere. His parents suggested seeking help from a psychiatric hospital, but this suggestion made John angry and agitated*.’

Respondents were asked to what extent they agreed to the following statements using a Likert scale. (1) John may harm himself or others. (2) John should be forced to go to a psychiatric hospital if he refuses to go. (3) John should be forced to take medications at the Psychiatric hospital when he is eventually taken there. (4) John should be tied up or mechanically restrained if he resists the medication or attempts to run away from the psychiatric hospital. (5) John should be locked up in a room alone if he resists the medication or attempts to run away from the psychiatric hospital.

The second section was an eight-item social distance scale used to assess an individual’s avoidance reaction directed toward PMHCs. The validity and reliability of the instrument have been proven in previous studies (Cates et al., 2011). The third section sought information on the sociodemographic characteristics of the respondents.

### Data analysis

The socio-demographic characteristics were summarized using descriptive statistics and reported as frequencies, percentages, means, and standard deviations. To compare the differences in the social distance score for different levels of acceptance for coercive measures, ANOVA tests were employed. Chi-squared tests were conducted to understand the associations between the socio-demographic characteristics and the acceptance of coercive measures. The *p*-values less than .05 were considered significant. Data were analyzed with SPSS v.25 software.

### Ethical considerations

The approval for the study was obtained from the Health Research and Ethics Committee of the Department of Clinical Pharmacy and Pharmacy Management, University of Nigeria Nsukka (CPPM/HREC/24/RES/0001). Informed consent for the study was obtained from each participant. They were assured of anonymity and confidentiality of the study data, and the right to withdraw consent if they wanted to.

## Results

More than half of the study respondents agreed that the vignette character should be forced to go to the hospital if he refuses to go (65%, *n* = 400) and he should be forced to take medications at the psychiatric hospital (55.1%, *n* = 339). The least accepted coercive measure was Isolation (28.8%, *n* = 177; Table 1).

**Table 1.** Agreement with coercive measures in the care of PMHCs.

To what extent do you agree with the following statements?	Disagree	Neutral	Agree	*M* ± *SD*
	*N* (%)	*N* (%)	*N* (%)	Score
John may harm himself or others.	43 (7.0)	38 (6.2)	534 (86.8)	2.79 ± 0.549
John should be forced to go to a psychiatric hospital if he refuses to go.	125 (20.3)	90 (14.6)	400 (65.0)	2.45 ± 0.809
John should be forced to take medications at the Psychiatric hospital when he is eventually taken there.	157 (25.5)	119 (19.3)	339 (55.1)	2.29 ± 0.849
John should be tied up or mechanically restrained if he resists the medication or attempts to run away from the psychiatric hospital.	285 (46.3)	108 (17.6)	222 (36.1)	1.89 ± 0.902
John should be locked up in a room alone if he resists the medication or attempts to run away from the psychiatric hospital.	351 (57.1)	87 (14.1)	177 (28.8)	1.72 ± 0.883

There were significant associations between social distance and acceptance of all the coercive measures explored (*p* < .05). Social distance score was highest among respondents who agreed that the vignette character should be isolated (24.023 ± 5.503; Table 2)

**Table 2.** Association between social distance and agreement with coercive measures.

To what extent do you agree with the following statements?	Disagree Mean total *SD* scores ± SD	Neutral Mean total *SD* scores ± SD	Agree Mean total *SD* scores ± SD	*F* (*p*-value)
John may harm himself or others.	22.53 ± 4.469	24.61 ± 3.759	22.44 ± 5.403	3.019 (.050)
John should be forced to go to a psychiatric hospital if he refuses to go.	21.28 ± 4.985	22.344 ± 5.591	23.04 ± 5.233	5.45 (.005)**
John should be forced to take medications at the Psychiatric hospital when he is eventually taken there.	21.29 ± 5.488	22.45 ± 4.588	23.21 ± 5.304	7.24 (.001) **
John should be tied up or mechanically restrained if he resists the medication or attempts to run away from the psychiatric hospital.	21.42 ± 5.053	22.66 ± 4.635	24.023 ± 5.503	15.92 (<.001)***
John should be locked up in a room alone if he resists the medication or attempts to run away from the psychiatric hospital.	21.49 ± 5.045	22.45 ± 4.707	24.79 ± 5.329	24.672 (<.001)***

SD scores: Social Distance scores.

### Sociodemographic differences in agreement with coercive measure

More males than females accepted isolation for the vignette character (30.7% vs. 26.1%, *p* < .05). There were no significant differences in the acceptance of forced hospitalization, forced medications, mechanical restraints, and isolation according to Age, Education, having the experience of a mental health condition or having a family member with a mental health condition (Table 3).

**Table 3.** Sociodemographic differences in agreement with coercive measures.

Variable	Forced hospitalisation				Forced medication				Mechanical restraints				Isolation			
	Disagree *N* (%)	Neutral *N* (%)	Agree *N* (%)	χ^2^	Disagree *N* (%)	Neutral *N* (%)	Agree	χ^2^	Disagree *N* (%)	Neutral *N* (%)	Agree *N* (%)	χ^2^	Disagree *N* (%)	Neutral *N* (%)	Agree *N* (%)	χ^2^
Gender				1.257				0.258				8.370*				6.086*
Male	70 (19.6)	57 (15.9)	231 (64.5)		89 (24.9)	71 (19.8)	198 (55.3)		155 (43.3)	76 (21.2)	127 (35.5)		190 (53.1)	58 (16.2)	110 (30.7)	
Female	55 (21.4)	33 (12.8)	169 (65.8)		68 (26.5)	48 (18.7)	141 (54.9)		130 (50.6)	32 (12.5)	95 (37.0)		161 (62.6)	29 (11.3)	67 (26.1)	
Age (years)				4.033				5.299				2.161				7.156
18–30	77 (18.5)	66 (15.9)	273 (65.6)		105 (25.2)	89 (21.4)	222 (53.4)		194 (46.6)	75 (18.0)	147 (35.3)		237 (57.0)	62 (14.9)	117 (28.1)	
31–40	31 (25.8)	14 (11.7)	75 (62.5)		34 (28.3)	20 (16.7)	66 (55.0)		57 (47.5)	22 (18.3)	41 (34.2)		77 (64.2)	14 (11.7)	29 (24.2)	
>40	17 (21.5)	10 (12.7)	52 (65.8)		18 (22.8)	10 (12.7)	51 (64.6)		34 (43.0)	11 (13.9)	34 (43.0)		37 (46.8)	11 (13.9)	31 (39.2)	
Education				9.297				4.495				1.783				3.758
<Bachelor’s degree	34 (30.1)	12 (10.6)	67 (59.3)		31 (27.4)	24 (21.2)	58 (51.3)		51 (45.1)	17 (15.0)	45 (39.8)		60 (53.1)	14 (12.4)	39 (34.5)	
Bachelor’s degree	79 (18.7)	65 (15.4)	278 (65.9)		105 (24.9)	86 (20.4)	231 (54.7)		195 (46.2)	75 (17.8)	152 (36.0)		240 (56.9)	64 (15.2)	118 (28.0)	
Postgraduate degree	12 (15.0)	13 (16.3)	55 (68.8)		21 (26.3)	9 (11.3)	50 (62.5)		39 (48.8)	16 (20.0)	25 (31.3)		51 (63.7)	9 (11.3)	20 (25.0)	
Religion				12.190				17.475**				5.736				10.802
Christianity	95 (23.6)	59 (14.7)	248 (61.7)		115 (28.6)	79 (19.7)	208 (51.7)		193 (48.0)	65 (16.2)	144 (35.8)		240 (59.7)	59 (14.7)	103 (25.6)	
Islam	26 (12.9)	30 (14.9)	146 (72.3)		36 (17.8)	38 (18.8)	128 (63.4)		87 (43.1)	40 (19.8)	75 (37.1)		106 (52.5)	25 (12.4)	71 (35.1)	
Traditional African Religion	1 (50.0)	0 (0.0)	1 (50.0)		2 (100)	0 (0)	0 (0)		2 (100)	0 (0)	0 (0)		2 (100.0)	0 (0)	0 (0)	
No religion	3 (33.3)	1 (11.1)	5 (55.6)		4 (44.4)	2 (22.2)	3 (33.3)		3 (33.3)	3 (33.3)	3 (33.3)		3 (33.3)	3 (33.3)	3 (33.3)	
Geopolitical Zone				19.197*				13.034				4.896				14.482
South-East	57 (26.4)	33 (15.3)	126 (58.3)		68 (31.5)	45 (20.8)	103 (47.7)		109 (50.5)	33 (15.3)	74 (34.3)		126 (58.3)	38 (17.6)	52 (24.1)	
South-West	22 (25.9)	12 (14.1)	51 (60.0)		23 (27.1)	14 (16.5)	48 (56.5)		40 (47.1)	16 (18.8)	29 (34.1)		55 (64.7)	8 (9.4)	22 (25.9)	
South-South	4 (13.8)	1 (3.4)	24 (82.8)		6 (20.7)	5 (17.2)	18 (62.1)		12 (41.4)	6 (20.7)	11 (37.9)		20 (69.0)	2 (6.9)	7 (24.1)	
North-Central	9 (18.0)	7 (14.0)	34 (68.0)		13 (26.0)	11 (22.0)	26 (52.0)		25 (50.0)	8 (16.0)	17 (34.0)		27 (54.0)	10 (20.0)	13 (26.0)	
North-West	4 (12.1)	3 (9.1)	26 (78.8)		6 (18.2)	9 (27.3)	18 (54.5)		12 (36.4)	6 (18.2)	15 (45.5)		18 (54.5)	4 (12.1)	11 (33.3)	
North-East	29 (14.4)	34 (16.8)	139 (68.8)		41 (20.3)	35 (17.3)	126 (62.4)		87 (43.1)	39 (19.3)	76 (37.6)		105 (52.0)	25 (12.4)	72 (35.6)	
Have a MHC				0.190				0.606				2.160				0.609
No	115 (20.3)	84 (14.8)	368 (64.9)		147 (25.9)	109 (19.2)	311 (54.9)		266 (46.9)	96 (16.9)	205 (36.2)		326 (57.5)	80 (14.1)	161 (28.4)	
Yes	10 (20.8)	6 (12.5)	32 (66.7)		10 (20.8)	10 (20.8)	28 (58.3)		19 (39.6)	12 (25.0)	17 (35.4)		25 (52.1)	7 (14.6)	16 (33.3)	
Have Family with MHC				1.208				0.905				5.710				5.422
No	78 (20.5)	60 (15.8)	242 (63.7)		102 (26.8)	72 (18.9)	206 (54.2)		166 (43.7)	63 (16.6)	151 (39.7)		203 (53.4)	58 (15.3)	119 (31.3)	
Yes	47 (20.0)	30 (12.8)	158 (67.2)		55 (23.4)	47 (20.0)	133 (56.6)		119 (50.6)	45 (19.1)	71 (30.2)		148 (63.0)	29 (12.3)	58 (24.7)	
May Harm Himself/Others				37.082**				11.174*				10.422*				2.663
Agree	89 (16.7)	77 (14.4)	368 (68.9)		125 (23.4)	105 (19.7)	304 (56.9)		236 (44.2)	94 (17.6)	204 (38.2)		304 (56.9)	72 (13.5)	158 (29.6)	
Neutral	16 (42.1)	5 (13.2)	17 (44.7)		14 (36.8)	5 (13.2)	19 (50.0)		21 (55.3)	6 (15.8)	11 (28.9)		22 (57.9)	8 (21.1)	8 (21.1)	
Disagree	20 (46.5)	8 (18.6)	15 (34.9)		18 (41.9)	9 (20.9)	16 (37.2)		28 (65.1)	8 (18.6)	7 (16.3)		25 (58.1)	7 (16.3)	11 (25.6)	

Bold indicates statistically significant Chi-square values.

* *p* < .05. **p < .01.

## Discussion

More than three-quarters of the study sample felt that the vignette character would harm himself or others and about 65% of them agreed that the character should be forced to go to a psychiatric hospital. A greater proportion of the respondents who agreed that the character would harm himself or others accepted that he should be forced to go to the psychiatric hospital if he refused. The level of agreement for forced admission in our study is higher than that reported in a Norwegian population survey, where 45.2% of participants supported involuntary admission for a young individual with newly developed symptoms of schizophrenia (Wynn et al., 2006). However, it is lower than the support for compulsory admission in a similar scenario involving first-episode paranoid delusions, observed in a cross-cultural study of psychiatrists, other professionals, and laypeople from four European countries: England, Germany, Hungary, and Switzerland. In that study, 75.8% of them agreed with compulsory hospitalization for a patient experiencing a first episode accompanied by social withdrawal (Steinert et al., 2005). Additionally, our findings are lower than those of a previous Swiss study, where over 70% of respondents supported compulsory admission (Lauber et al., 2002). It is important to note that the Swiss study did not use case-specific vignettes. It has been suggested that the use of case vignettes versus general statements can significantly impact public attitudes toward coercive measures, potentially contributing to differences in acceptance levels (Steiger et al., 2023). In addition to methodological, legislative, and other contextual differences from the studies cited, the preference for spiritual and traditional healers for mental health conditions among Nigerians (Jidong et al., 2021; Okafor et al., 2022) may also explain the lower acceptance of forced psychiatric hospitalization for the vignette character among respondents in this study. Steinert et al. (2005) suggest that the higher level of support for involuntary admission among English respondents might be reflective of trust in the system attributed to their lack of experience with totalitarian dictatorship and abuse. Following this argument, the relatively lower support for forced hospitalization in our study could reflect Nigeria’s history of totalitarian dictatorships and ongoing experiences of human rights violations.

It is worthwhile to note that Nigeria’s Mental Health Act (National Mental Health Act, 2021) requires evidence of a mental disorder and a serious likelihood of imminent harm to oneself or others due to the mental health condition for involuntary admissions to take place. Alternatively, there must be evidence that the mental health condition is so severe that failure to admit the person would likely hinder the provision of appropriate treatment, which can only be given in a facility following the principle of the least restrictive alternative. The high levels of agreement for the forced hospitalization of the vignette character, who was not described as violent or potentially dangerous, possibly indicate a lack of awareness of the criteria for forced hospitalization in Nigeria’s National Mental Health Act 2021. This could be because the mental health legislation outlining such criteria was only passed in January 2023 for the first time in the country`s history, making it plausible that the public is not well acquainted with its provisions. On the other hand, it could also reflect high levels of concern and care expected from parents or relatives of PMHCs, considering that forced admission is not a free service in Nigeria. It may be a case of forcing the vignette character into treatment early to avoid significant deterioration in their condition. Research on public opinion about using coercive measures in mental health care is limited, especially in developing countries. However, studies conducted among clinicians in India suggest that they generally view coercion positively (Gowda et al., 2019; Raveesh et al., 2016). This attitude may reflect broader public opinion. In the context of limited resources, many see coercion as providing necessary care, protecting patients and others, and minimizing harm.

Isolation was the least accepted coercive measure for the vignette character in the psychiatric hospital, with fewer than one-third of respondents approving of it. In communitarian cultures like Nigeria, isolation is viewed as one of the harshest punishments because it entirely deprives individuals of social interaction. Consequently, it is rarely practiced, both due to its severity and the lack of available space (Aluh, Ayilara, et al., 2023). This finding aligns with the study conducted in Switzerland, where approval for seclusion was also the lowest, indicating that seclusion was seen as the most severe coercive measure (Steiger et al., 2023). The desire for social distance, which indicates stigma, was associated with the approval of all the coercive measures explored in the current study. This contrasts with a previous study among the Swiss population, where social distance was not linked to approval of involuntary hospitalization (Steiger et al., 2022). The social distance scores seemed to progressively increase with the severity of the coercive measures, reaching their highest among respondents who agreed that the vignette character should be isolated. A significantly higher proportion of males than females accepted isolation for the vignette character, in keeping with extant literature that suggests that isolation is particularly more painful and traumatizing for females (Smith et al., 2023).

Lower support for coercive measures has been linked to knowing someone with a mental health condition or having firsthand experience with psychiatric treatment (Steiger et al., 2023). In the current study, having a family member or a personal history of mental health conditions was not associated with acceptance of coercive measures, consistent with the findings of Steinert et al. (2005). This lack of association may be attributed to the presence of internalized stigma among individuals with mental health conditions as well as their relatives. In the context of our study, high levels of internalized stigma have been reported (Adewuya et al., 2011; Akinjola et al., 2021), which could explain why participants with personal or familial experiences of mental health conditions did not show significantly lower acceptance of coercive measures

Additionally, there was no significant association between acceptance of coercive measures and educational attainment, unlike previous studies which reported that reluctance to accept infringements on the rights of PMHCs increased with higher levels of educational attainment (Angermeyer et al., 2014). Although there were no significant differences in the acceptance of forced hospitalization and forced medication, the proportion of respondents agreeing with these measures increased with higher educational attainment. Conversely, support for mechanical restraints and isolation decreased among more educated individuals, suggesting that they were less inclined to endorse harsher coercive measures.

### Strengths and limitations

The use of case-specific vignettes is both a strength and a limitation. On the one hand, clinical vignettes describing detailed patient cases have been reported to allow respondents to empathize with the case and consider the pros and cons of applying coercive measures, in contrast to general questions about the acceptance of coercive measures in psychiatry (Steiger et al., 2023). On the other hand, the extent to which these subjective responses based on a case vignette can be translated into real-world scenarios is unclear. The concept of desired social distance is used in this study to operationalize stigmatization. It remains uncertain to what extent this behavioral intention will manifest as actual behavior. The online questionnaire-based approach may have facilitated the participation of individuals with a relatively high level of education. The survey was completed by people with lived experience of mental health conditions, staff members from mental health services and other members of the public. However, without a specific question about the profession, it was not possible to directly compare responses among the groups. Furthermore, the convenience sampling makes it unclear how broadly our findings can be generalized to the overall population, particularly to non-literate people in rural areas.

## Conclusion

Culturally sensitive mental health policies are urgently needed in Nigeria, and indeed most developing countries to reduce coercive psychiatric practices. While the mental health laws provide a framework for achieving this, their implementation requires multifaceted approaches. Prioritizing mental health literacy, stigma reduction, and service user empowerment is crucial in addition to service reform, especially so in Nigeria`s context. Deep-seated social prejudices hinder efforts to reduce coercion, so actively addressing misconceptions is essential. Service users, with their firsthand experience, are best placed to advocate for their rights and drive change. Mental health professionals must promote ethical practices and human rights through ongoing training. Collaboration with service users is key to fostering a culture of respect. Policymakers must invest in mental health systems, implement policies protecting patient rights, and allocate resources to community-based support to minimize coercion in mental health care.

## References

[bibr1-00207640241296050] Adewuya A. O. Owoeye A. O. Erinfolami A. O. Ola B. A. (2011). Correlates of self-stigma among outpatients with mental illness in Lagos, Nigeria. The International Journal of Social Psychiatry, 57(4), 418–427. 10.1177/0020764010363522 20354068

[bibr2-00207640241296050] African Union. (2018). Protocol to the African charter on human and peoples’ rights on the rights of persons with disabilities in Africa. Retrieved June 30, 2023, from https://au.int/en/treaties/protocol-african-charter-human-and-peoples-rights-rights-persons-disabilities-africa

[bibr3-00207640241296050] Akinjola O. Lawal R. A. Abayomi M. O. Adeosun I. I. Adefemi A. A. Adegbaju D. A. Agbir T. M. (2021). Self-stigma in Patients with Schizophrenia in a Psychiatry Hospital in Lagos, Nigeria. International Neuropsychiatric Disease Journal, 15(4), 37–50. 10.9734/INDJ/2021/V15I430162

[bibr4-00207640241296050] Aluh D. O. Ayilara O. Onu J. U. Grigaitė U. Pedrosa B. Santos-Dias M. Cardoso G. Caldas-de-Almeida J. M. (2022). Experiences and perceptions of coercive practices in mental health care among service users in Nigeria: A qualitative study. International Journal of Mental Health Systems, 16(1), 1–11. 10.1186/S13033-022-00565-4 36424651 PMC9694572

[bibr5-00207640241296050] Aluh D. O. Ayilara O. Onu J. U. Pedrosa B. Silva M. Grigaitė U. Santos-Dias M. Cardoso G. Caldas-de-Almeida J. M. (2023). Use of coercion in mental healthcare services in Nigeria: Service providers’ perspective. . Journal of Mental Health, 33(1), 75–83. 10.1080/09638237.2023.2182426 36850036

[bibr6-00207640241296050] Aluh D. O. Dim O. F. Anene-Okeke C. G. (2018). Mental health literacy among Nigerian teachers. Asia-Pacific Psychiatry, 10(4), Article e12329. 10.1111/appy.12329 30175891

[bibr7-00207640241296050] Aluh D. O. Okonta M. J. Odili V. U. (2019). Cross-sectional survey of mental health literacy among undergraduate students of the University of Nigeria. BMJ Open, 9(9), Article e028913. 10.1136/bmjopen-2019-028913 PMC6747681 31515420

[bibr8-00207640241296050] Aluh D. O. Onu J. U. Almeida J. M. C. (2023). A new era for mental health care in Nigeria. The Lancet Psychiatry, 10(5), 310–311. 10.1016/S2215-0366(23)00071-8 36921611

[bibr9-00207640241296050] Angermeyer M. C. Matschinger H. Schomerus G. (2014). Attitudes of the German public to restrictions on persons with mental illness in 1993 and 2011. Epidemiology and Psychiatric Sciences, 23(3), 263–270. 10.1017/S2045796014000183 24703571 PMC6998258

[bibr10-00207640241296050] Audu I. A. Idris S. H. Olisah V. O. Sheikh T. L. (2013). Stigmatization of people with mental illness among inhabitants of a rural community in northern Nigeria. International Journal of Social Psychiatry, 59(1), 55–60. 10.1177/0020764011423180 22131198

[bibr11-00207640241296050] Cates M. E. Bright J. T. Kamei H. Woolley T. W. (2011). Attitudes of Japanese pharmacy students toward mental illness. Pharmacy Education, 11. https://pharmacyeducation.fip.org/pharmacyeducation/article/view/330

[bibr12-00207640241296050] Cochran W. G. (1963). Sampling techniques. Wiley.

[bibr13-00207640241296050] Discrimination Against Persons with Disabilities (Prohibition) Act. (2018).

[bibr14-00207640241296050] Gowda G. Lepping P. Ray S. Noorthoorn E. Nanjegowda R. Kumar C. Math S. (2019). Clinician attitude and perspective on the use of coercive measures in clinical practice from tertiary care mental health establishment – A cross-sectional study. Indian Journal of Psychiatry, 61(2), 151–155. 10.4103/PSYCHIATRY.INDIANJPSYCHIATRY_336_18 PMC6425791 30992609

[bibr15-00207640241296050] Gureje O. Lasebikan V. O. Ephraim-Oluwanuga O. Olley B. O. Kola L. (2005). Community study of knowledge of and attitude to mental illness in Nigeria. The British Journal of Psychiatry: The Journal of Mental Science, 186, 436–441. 10.1192/BJP.186.5.436 15863750

[bibr16-00207640241296050] Herrman H. Allan J. Galderisi S. Javed A. Rodrigues M. (2022). Alternatives to coercion in mental health care: WPA Position Statement and Call to Action. World Psychiatry, 21(1), 159–160. 10.1002/WPS.20950 35015368 PMC8751569

[bibr17-00207640241296050] Human Rights Watch. (2019). Nigeria: People with mental health conditions chained, abused. Retrieved June 20, 2024, from https://www.hrw.org/news/2019/11/11/nigeria-people-mental-health-conditions-chained-abused

[bibr18-00207640241296050] Human Rights Watch. (2024). World report 2024: Nigeria. Retrieved May 31, 2024, from https://www.hrw.org/world-report/2024/country-chapters/nigeria

[bibr19-00207640241296050] James B. O. Omoaregba J. O. Okogbenin E. O. (2012). Stigmatising attitudes towards persons with mental illness: a survey of medical students Southern Nigeria. Mental Illness, 4(8), 32–34. 10.4081/mi.2012.e8 PMC4253366 25478110

[bibr20-00207640241296050] Jidong D. E. Bailey D. Sodi T. Gibson L. Sawadogo N. Ikhile D. Musoke D. Madhombiro M. Mbah M. (2021). Nigerian cultural beliefs about mental health conditions and traditional healing: A qualitative study. Journal of Mental Health Training, Education and Practice, 16(4), 285–299. 10.1108/JMHTEP-08-2020-0057/FULL/XML

[bibr21-00207640241296050] Jorm A. F. Korten A. E. Jacomb P. A. Christensen H. Rodgers B. Pollitt P. (1997). “Mental health literacy”: a survey of the public’s ability to recognise mental disorders and their beliefs about the effectiveness of treatment. The Medical Journal of Australia, 166(4), 182–186. 10.5694/J.1326-5377.1997.TB140071.X 9066546

[bibr22-00207640241296050] Jorm A. F. Reavley N. J. Ross A. M. (2012). Belief in the dangerousness of people with mental disorders: a review. The Australian and New Zealand Journal of Psychiatry, 46(11), 1029–1045. 10.1177/0004867412442406 22422995

[bibr23-00207640241296050] Lauber C. Nordt C. Falcato L. Rössler W. (2002). Public attitude to compulsory admission of mentally ill people. Acta Psychiatrica Scandinavica, 105(5), 385–389. 10.1034/J.1600-0447.2002.1O267.X 11942946

[bibr24-00207640241296050] Lunacy Act. (1958).

[bibr25-00207640241296050] National Mental Health Act. (2021).

[bibr26-00207640241296050] Okafor I. P. Oyewale D. V. Ohazurike C. Ogunyemi A. O. (2022). Role of traditional beliefs in the knowledge and perceptions of mental health and illness amongst rural-dwelling women in western Nigeria. African Journal of Primary Health Care & Family Medicine, 14(1), e1–e8. 10.4102/PHCFM.V14I1.3547 PMC9575354 36226933

[bibr27-00207640241296050] Raveesh B. Pathare S. Noorthoorn E. Gowda G. Lepping P. Bunders-Aelen J. (2016). Staff and caregiver attitude to coercion in India. Indian Journal of Psychiatry, 58(Suppl. 2), S221–S229. 10.4103/0019-5545.196847 PMC5282619 28216773

[bibr28-00207640241296050] Ronzoni P. Dogra N. Omigbodun O. Bella T. Atitola O. (2010). Stigmatization of mental illness among Nigerian schoolchildren. The International Journal of Social Psychiatry, 56(5), 507–514. 10.1177/0020764009341230 19651693

[bibr29-00207640241296050] Sheikh T. L. Adekeye O. Olisah V. O. Mohammed A. (2015). Stigmatisation of mental illness among employees of a Northern Nigerian University. Nigerian Medical Journal, 56(4), 244–248. 10.4103/0300-1652.169697 26759507 PMC4697210

[bibr30-00207640241296050] Simmons B. A. (2014). The future of the human rights movement. Columbia University.

[bibr31-00207640241296050] Smith L. R. Ingel S. Rudes D. S. (2023). “Like an animal”: The well-being of women living in restricted housing units. Health & Justice, 11(1), 15. 10.1186/S40352-023-00215-Y 36884088 PMC9993580

[bibr32-00207640241296050] Steiger S. Moeller J. Sowislo J. F. Lieb R. Lang U. E. Huber C. G. (2022). Approval of coercion in psychiatry in public perception and the role of stigmatization. Frontiers in Psychiatry, 12, Article 2513. 10.3389/FPSYT.2021.819573/BIBTEX PMC8777226 35069299

[bibr33-00207640241296050] Steiger S. Moeller J. Sowislo J. F. Lieb R. Lang U. E. Huber C. G. (2023). General and case-specific approval of coercion in psychiatry in the public opinion. International Journal of Environmental Research and Public Health, 20(3), Article 2081. 10.3390/IJERPH20032081 PMC9916390 36767450

[bibr34-00207640241296050] Steinert T. Lepping P. Baranyai R. Hoffmann M. Leherr H. (2005). Compulsory admission and treatment in schizophrenia: a study of ethical attitudes in four European countries. Social Psychiatry and Psychiatric Epidemiology, 40(8), 635–641. 10.1007/S00127-005-0929-7 16133746

[bibr35-00207640241296050] Ubaka C. M. Chikezie C. M. Amorha K. C. Ukwe C. V. (2018). Health Professionals’ Stigma towards the Psychiatric Ill in Nigeria. Ethiopian Journal of Health Sciences, 28(4), 483–494. 10.4314/ejhs.v28i4.14 30607061 PMC6308735

[bibr36-00207640241296050] Wynn R. Myklebust L. H. Bratlid T. (2006). Attitudes to coercion among health-care workers and the general public in Norway. Journal of Psychiatric Intensive Care, 2(1), 31–37. 10.1017/S1742646406000252

